# Altered mucosal immune response after acute lung injury in a murine model of Ataxia Telangiectasia

**DOI:** 10.1186/1471-2466-14-93

**Published:** 2014-05-29

**Authors:** Olaf Eickmeier, Su Youn Kim, Eva Herrmann, Constanze Döring, Ruth Duecker, Sandra Voss, Sibylle Wehner, Christoph Hölscher, Julia Pietzner, Stefan Zielen, Ralf Schubert

**Affiliations:** 1 Pediatric Pulmonology, Allergy and Cystic Fibrosis, Johann Wolfgang Goethe- University, Theodor-Stern-Kai 7, Frankfurt D-60590, Germany; 2 Institute of Biostatistics and Mathematical Modeling, Johann Wolfgang Goethe-University, Frankfurt, Germany; 3 Senckenberg Institute of Pathology, Johann Wolfgang Goethe- University, Frankfurt, Germany; 4 Pediatric Hematology and Oncology, Johann Wolfgang Goethe- University, Frankfurt, Germany; 5 Division of Infection Immunology, Research Center Borstel, Borstel, Germany

**Keywords:** Ataxia telangiectasia, ATM, Acute lung injury, Inflammation

## Abstract

**Background:**

Ataxia telangiectasia (A-T) is a rare but devastating and progressive disorder characterized by cerebellar dysfunction, lymphoreticular malignancies and recurrent sinopulmonary infections. In A-T, disease of the respiratory system causes significant morbidity and is a frequent cause of death.

**Methods:**

We used a self-limited murine model of hydrochloric acid-induced acute lung injury (ALI) to determine the inflammatory answer due to mucosal injury in *Atm* (A-T mutated)- deficient mice (*Atm*^-/-^).

**Results:**

ATM deficiency increased peak lung inflammation as demonstrated by bronchoalveolar lavage fluid (BALF) neutrophils and lymphocytes and increased levels of BALF pro-inflammatory cytokines (e.g. IL-6, TNF). Furthermore, bronchial epithelial damage after ALI was increased in *Atm*^-/-^ mice. ATM deficiency increased airway resistance and tissue compliance before ALI was performed.

**Conclusions:**

Together, these findings indicate that ATM plays a key role in inflammatory response after airway mucosal injury.

## Background

Ataxia telangiectasia (A-T) is a severe autosomal recessive disorder that is caused by mutations in the ATM gene which codes for ataxia telangiectasia mutated (ATM), a pleiotropic kinase involved in DNA double-strand break recognition, activation of DNA repair proteins, and signaling in cell cycle checkpoint control [1-5]. This rare human disease encompasses defects in T- and B- cell maturation, cerebellar degeneration, radiosensitivity, and increased susceptibility to malignancies, especially lymphomas [6-11]. The majority of patients suffers from IgA and IgG subclass deficiency and has impaired antibody responses to pneumococcal antigens [12,13]. Immunodeficiency is considered to be the primary factor predisposing these patients to the development of chronic respiratory tract infections. The 20 year survival rate is 53.4%, and death is predominantly caused by lung failure and cancer. The prognosis has not changed since 1954 [14]. *Atm-* deficient (Atm^-/-^) mice exhibit similar defects as A-T patients [9,15-19] and have been useful for studying A-T, mechanisms of DNA damage responses, and oncogenesis.

Lung disease is common in patients with A-T and often progresses with age and neurological decline [20]. Three major types of lung disease are generally recognized in A-T patients: Recurrent sinopulmonary infections and bronchiectasis, interstitial lung disease (ILD)/ pulmonary fibrosis and lung disease associated with neuromuscular deficits due to bulbar and spinal system impairment [20-24]. An evaluation of a series of patients with A-T showed an increased frequency and severity of signs and symptoms of impaired deglutition and an association between dysphagia, aspiration and pulmonary disease of these patients was confirmed [25]. Clarifying the impact of cellular and clinical factors on A-T lung disease and its progression may help with discovery and development of therapeutic interventions.

Aspiration pneumonitis is one of the leading causes of Acute Lung Injury (ALI)/ Acute Respiratory Distress Syndrome (ARDS) that is in most instances self-limited, suggesting the existence of endogenous, host protective mechanisms [26]. Here, in a non-lethal model of ALI [27,28], *Atm*^-/-^mice were challenged with intratracheal application of hydrochloric acid (HCl) in order to examine if host protective mechanisms are disturbed after mucosal injury.

## Methods

### Animal model

All mice were maintained under specific pathogen-free conditions and were checked daily. All studies were reviewed and approved by the German Animal Subjects Committee (Gen.Nr.F133/10). We used 10–12 week-old, male *Atm*^-/-^ mice (*Atm*^*tm1Awb*^), in a 129SvEv background [18], kindly provided by A. Wynshaw-Boris, School of Medicine, University California San Diego, USA, were used as animal model. Weights of the Atm^-/-^ mice (20 g ±0.4 (n ≥ 5); mean ± S.E.M.) were different from the wild type animals (24 g ± 0.8; (n ≥ 5); mean ± S.E.M.; p < 0.01).

### Acid-initiated acute lung injury (ALI) and treatment with AT-RVD1

Hydrochloric acid (0.1 N HCl, pH 1.5, 50 μl, endotoxin free; Sigma-Aldrich) was instilled selectively into the left mainstem bronchus of anesthetized mice via a 24-gauge angiocatheter inserted intratracheally. Due to high mortality rate in the *Atm*^-/-^ mice, the experimental setting was changed to instillation of 1 μl/g body weight of 0.1 N HCl (pH 1.5) into the left mainstem bronchus of anesthetized mice. At different time points (24 hours and 1 week) after acid instillation, bilateral bronchoalveolar lavage (BAL) was performed with 2 aliquots of 1 ml of PBS plus 0.6 mM EDTA. The total cell counts and leukocyte differential in BAL fluids (BALFs) were determined as previously described [28]. Briefly, total cells in BALFs were counted using a hemocytometer, and differential cell counts were determined after cytospin using Wright-Giemsa staining.

### Histology and immunohistochemistry

Mice were anesthetized with an intraperitoneal Ketamin–Rompun mixture (20% Ketamin, CuraMED GmbH, Karlsruhe, Germany; 8% Rompun, Bayer Vital GmbH, Leverkusen,Germany) injection. They were perfused transcardially with 4% paraformaldehyde in PBS. Lung tissue sections were prepared from fixed, paraffin-embedded organs and stained with either hematoxylin and eosin (H&E) or a special mucous staining, namely Alcian blue and van Giesona (ABvG).

### Measurement of lung mechanics

For measurement of tissue elastance and lung resistance, mice were anesthetized and mechanically ventilated with a flexiVent small animal ventilator (SCIREQ, Montreal, Canada). Lung mechanics were determined in anesthetized, ventilated animals.

### Mediator levels in BALFs

Select cytokines and chemokines were measured in aliquots of BALF by cytometric bead array as described [29].

### Statistical analysis

Data are expressed as mean values ± S.E.M. unless otherwise indicated. A two factorial analysis of variance of the logarithmized cell counts with interactions was used discriminate between differences caused by experimental conditions over time and those caused by experimental groups with the corresponding F test. Gauss distribution of the residuals was checked with the Shapiro-Wilk test.

Parametric or nonparametric analysis of variance was used to determine significance for differences between more than 2 groups as appropriate. For analyses between 2 groups, cohorts were compared by Mann–Whitney-U- test. Significance was determined with P values ≤ 0.05. Statistics were performed using GraphPad Prism 5 for Windows (San Diego, CA, USA).

## Results

### *Atm*^-/-^ deficient mice show elevated sensitivity to airway mucosal injury

**Figure 1:**
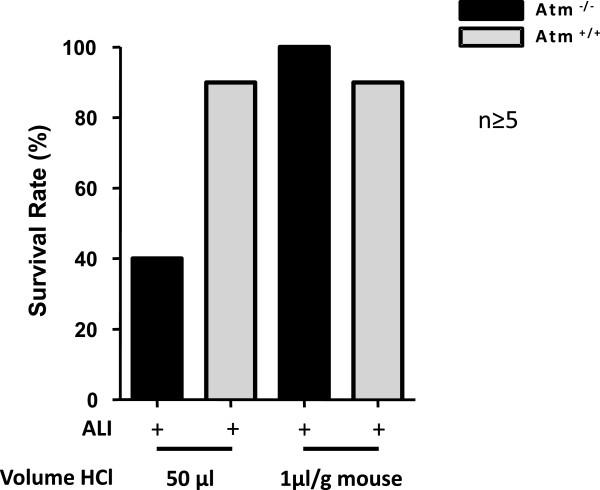
**Survival rate after ALI in *Atm***^**-/- **^**and healthy control mice with different dosing strategies.** Survival rate 24 hours after ALI in *Atm*^-/-^ (black bars) and control mice (grey bars) after instillation of 50 μl HCl in comparison to bodyweight adjusted (1 μl/ g mouse) HCl instillation.

In an *in vivo* setting we examined the role of ATM in the pathogenesis of airway mucosal injury due to ALI. In order to measure the inflammatory and resolutional response after acute lung injury, 50 μl of hydrochloric acid (0.1 N HCl, pH 1.5) was instilled into the left mainstem bronchus. Remarkably, *Atm*^-/-^mice showed elevated mortality due to airway HCl exposure in a defined mild injury experimental setting [27,28] in comparison to healthy control mice (Figure 1). A mortality rate of 60% in the *Atm*^-/-^group (n > 5) in comparison to a mortality rate of 10% in the control group (n > 5) was the reason for changing the injury protocol. So we adjusted the acid dosage to the body weight of the mice. In particular, we injected 1 μl/g body weight of hydrochloric acid into the left mainstem bronchus and thus increased the survival rate in the *Atm*^-/-^group to 100% (Figure 1).

### Defective ATM increases leukocyte recruitment after airway mucosal injury

**Figure 2:**
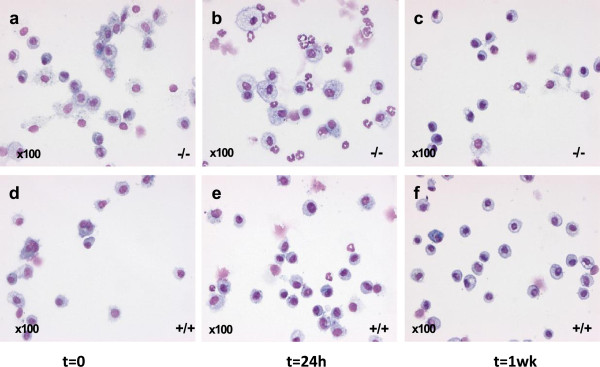
**Leukocyte clearance after airway mucosal injury in *Atm***^**-/- **^**and healthy control mice.** BALF cell differentiation before ALI between *Atm*^-/-^**(a)** and control mice **(d)**. *Atm*^-/-^ mice show decreased PMNs clearance **(b)** 24 hours after ALI representing increased inflammation and disturbed resolution compared to control mice **(e)**. These differences have resolved 1 week after ALI **(c** and **f)**. *Atm*^-/-^ mice **(a-c)**, *Atm*^+/+^mice **(d-e)**. Original magnification x100.

**Figure 3:**
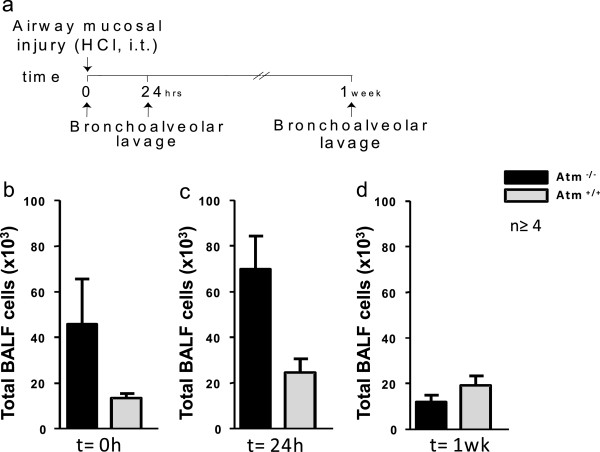
**ATM deficiency increases leukocyte recruitment after airway mucosal injury. (a)***Atm*^-/-^ mice or control mice underwent acute lung injury (ALI) by intratracheal (i.t.) instillation of HCl and bronchoalveolar lavage (BAL) was performed at different time points. Before ALI **(b)**, 24 hours after ALI **(c)** and one week after ALI **(d)** BAL was performed and total cells **(b-d)** were enumerated (see Methods). Values represent the mean ± S.E.M. for *n* ≥ 4 mice.

**Figure 4:**
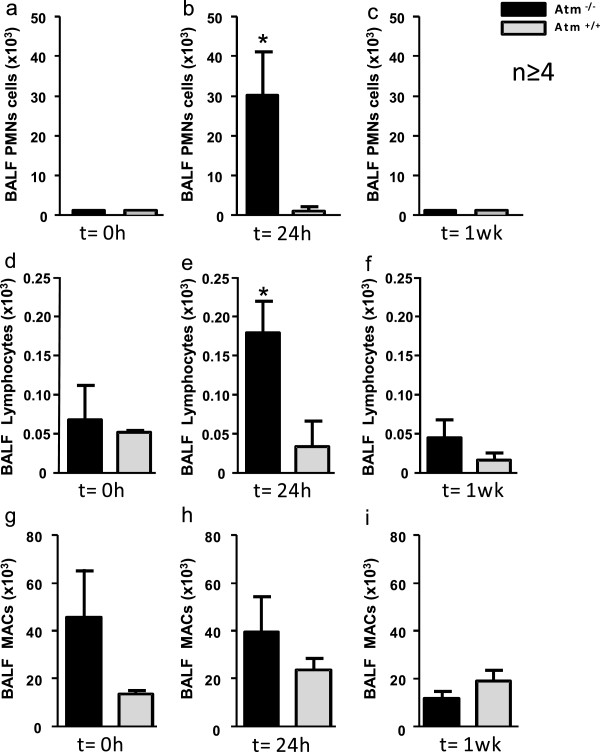
**ATM deficiency increases polymorphonuclear leukocytes (PMNs) and lymphoytes recruitment after airway mucosal injury.***Atm*^-/-^ or control mice underwent acute lung injury (ALI) by i.t. instillation of HCl and bronchoalveolar lavage (BAL) was performed at different time points. Before ALI **(a, d, g)**, 24 hours after ALI **(b, e, h)** and one week after ALI **(c, f, i)** BAL was performed and differential cell count for PMNs **(a-c)**, lymphocytes **(d-f)** and macrophages (MACs) **(g-i)** was done. Values represent the mean ± S.E.M. for *n ≥* 4 mice. * *P* < 0.05 vs. vehicle group.

To determine the effects of ATM on host responses to mucosal injury, BAL was performed on different time points after instillation of hydrochloric acid (0.1 N HCl, pH 1.5) into the left mainstem bronchus (Figure 2a-f and Figure 3a). There was a significant change in total BALF cell numbers after ALI (p = 0.017). Total BALF cell numbers 24 hours after ALI in *Atm*^-/-^mice (70.130 total BALF cells ± 14.630 (n = 5); mean ± S.E.M.) were larger than in healthy control mice (24.840 total BALF cells ± 5898; (n = 4); mean ± S.E.M.) (Figure 3b-d) but this difference was not significant in two-factorial ANOVA. There was no significant difference in total leukocyte and differential cell count between *Atm*^-/-^ mice before (Figure 3b and Figure 4a,d,g) or one week after (Figure 3d and Figure 4c,f,i) ALI. However, 24 hours after injury *Atm*^-/-^mice showed increased BALF neutrophils (PMNs) (30.300 PMNs ± 11.210 (n = 5); mean ± S.E.M.) as compared to healthy control mice (1.220 PMNs ± 647 (n = 4); mean ± S.E.M.) (Figure 4b). Difference over time after injury as well as interactions between *Atm*^-/-^ mice and control mice were significant here (p < 0.001 and p = 0.012, respectively). BALF lymphocytes were also increased in *Atm*^-/-^mice (179 lymphocytes ± 43; (n = 5); mean ± S.E.M.) and in healthy control mice (34 lymphocytes ± 34, n = 4, mean ± S.E.M.; *p* = 0.042 for time-effect in two- factorial ANOVA) (Figure 4e). There were no significant differences in the number of macrophages 24 hours after ALI in *Atm*^-/-^mice compared to control mice (Figure 4h).

### ATM deficiency impacts the lung histopathological changes after acid- initiated acute lung injury (ALI)

**Figure 5:**
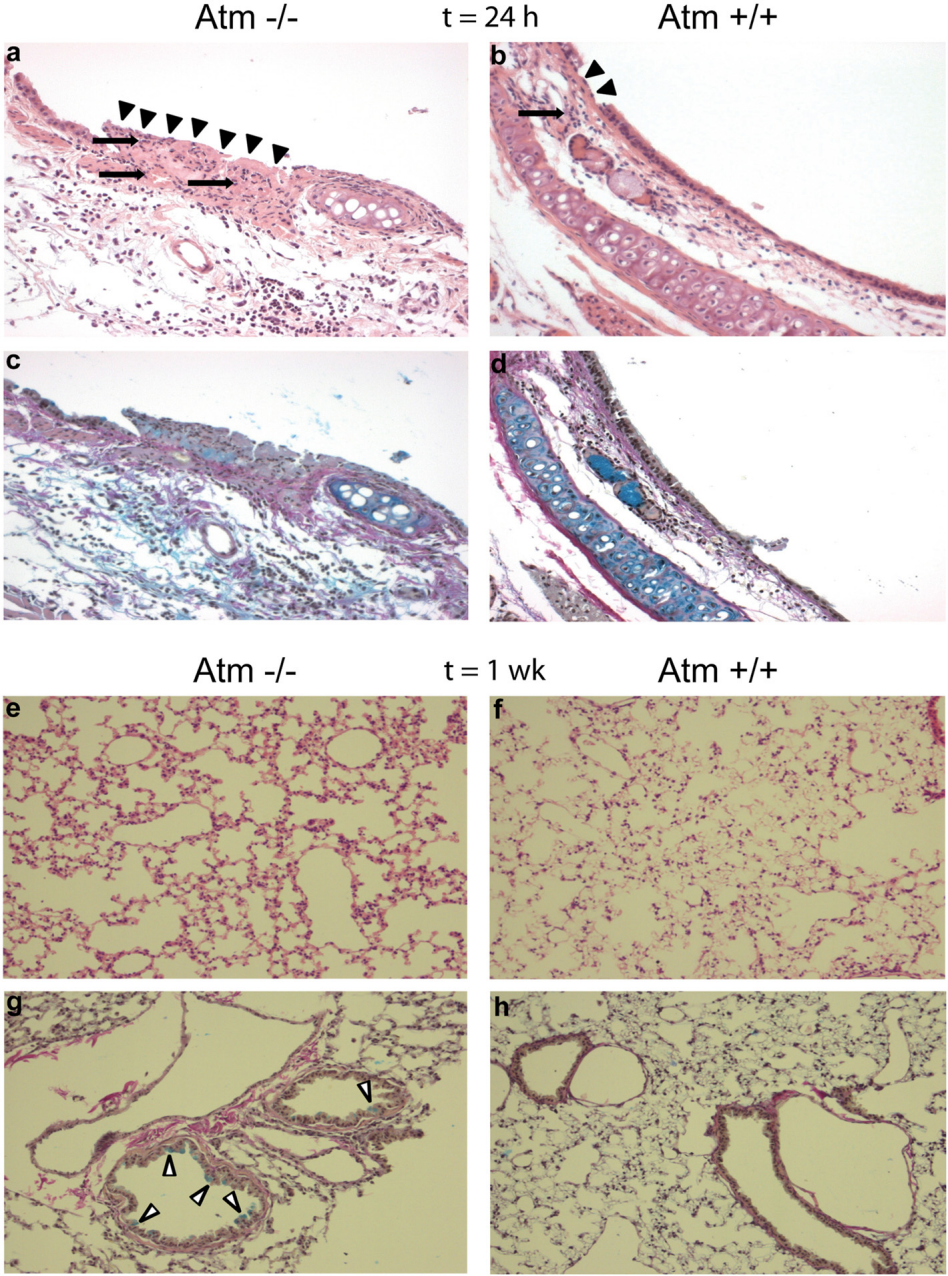
**ATM deficiency impacts the lung histopathological changes after acid –initiated acute lung injury (ALI).** Lung tissue sections were obtained 24 hours after **(a-d)** and one week after **(e-h)** ALI from *Atm*^-/-^**(a, c, e, g)** and control mice **(b, d, f, h)**. Hematoxylin and eosin (H&E) **(a, b, e, f)** or ABvG **(c, d, g, h)**. Results are representative of *n* = 3. Black arrowheads, epithelial cell disrupture; white arrowheads, mucus; black arrows, inflammatory cells. Original magnification x200.

Acute lung injury due to HCl acid instillation led to increased epithelial disruption 24 hours after exposure to acid in *Atm*^-/-^mice relative to control mice (Figure 5a-d). One week after mucosal injury *Atm*^-/-^mice showed hyperemic lung tissue (Figure 5e) and increased mucus production (Figure 5g) in comparison to control mice (Figure 5f and h).

### ATM deficient mice show elevated airway resistance and decreased tissue compliance

**Figure 6:**
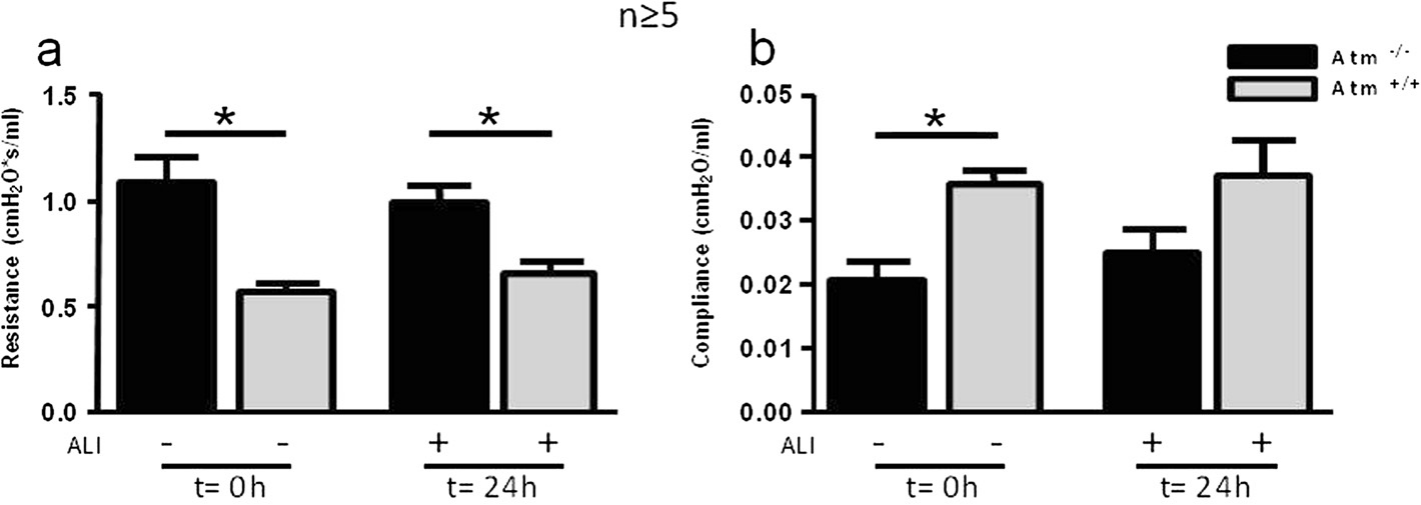
**Elevation in lung resistance and increased tissue compliance in *Atm***^**-/- **^**mice before and after mucosal injury.** Using a flexiVent mouse ventilator, **(a)** tissue resistance and **(b)** lung compliance were determined before and 24 hours after acid injury. Values represent the mean ± S.E.M. for n ≥ 5 mice. *P < 0.05 vs. control group.

To investigate whether ATM deficiency had a measurable effect on lung mechanics, we determined lung function in mechanically-ventilated, anesthetized mice. Possibly because of the unilateral and mild nature of the ALI in this model, marked changes in airway resistance (Figure 6a) and tissue compliance (Figure 6b) were not observed and no significant differences were evident with ATM deficiency 24 hours after ALI (Figure 6a and b). Of interest, the *Atm*^-/-^mice showed increased lung resistance (1.1 cmH_2_O*s/ml ± 0.12, (n = 5)) and decreased tissue compliance (0.02 cmH_2_O*/ml ± 0.003, (n = 5)) before mucosal damage was performed in comparison to control mice (resistance: 0.57 cmH_2_O*s/ml ± 0.03, mean ± S.E.M., (n = 6), *p* < 0.001; compliance (0.04 cmH_2_O/ml ± 0.002, mean ± S.E.M., (n = 6), *p* < 0.005) (Figure 6a and b).

### ATM deficiency enhances pro-inflammatory mediator release after ALI

**Figure 7:**
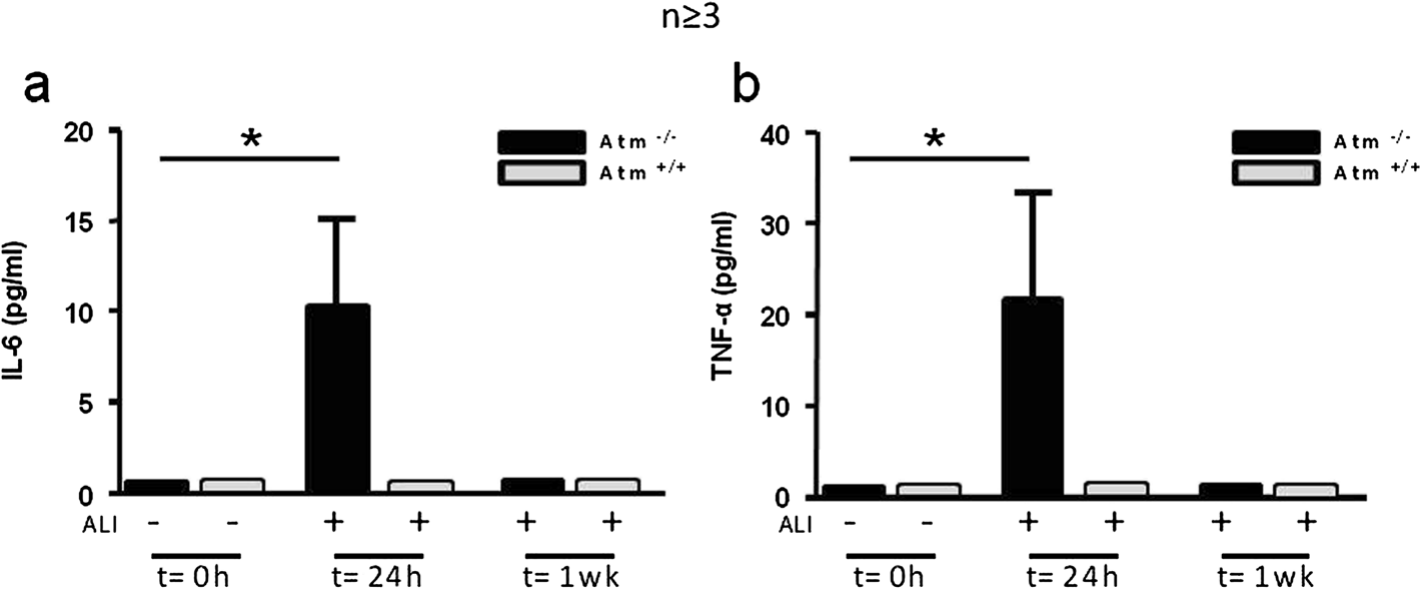
**Impact of ATM deficiency on inflammatory mediator levels after ALI.** Aliquots of BALFs obtained before ALI, 24 hours and one week after ALI were analyzed by cytometric bead array **(a, b)**. Values represent the mean ± S.E.M. for n ≥ 3 mice. *P < 0.05 vs. control group.

To identify anti-inflammatory or pro-resolving mechanisms for ATM in ALI, BALF levels of several cytokines were determined by cytometric bead array. BALF IL-6 (Figure 7a) and TNF-α (Figure 7b) was significantly increased in Atm^-/-^ mice 24 hours after ALI. BALF levels of other mediators did not increase with Atm deficiency relative to healthy control mice. No significant changes were observed in BALF for IL-10, IL-4, IL-12p40, IL-17A and IFN-γ (data not shown).

## Discussion

Lung disease is a common feature in patients with A-T and often progresses with age and neurological decline [20]. Diseases of the respiratory system cause significant morbidity and are a frequent factor or cause of death in the A-T population [20,30-32]. Several factors contribute to the increased susceptibility to respiratory infections. Immunodeficiency may be one, but not the only, etiology for lung disease in A-T. Low levels of pneumococcal antibodies and diminished levels of IgG subclasses are hallmarks of A-T and associated with respiratory tract infection frequency. However, not all respiratory tract infections in A-T are solely due to immunodeficiency [13,32]. Abnormal injury repair, premature aging, systemic inflammation, and oxidative stress are confirmed to contribute to the pathophysiology and disease progression in A-T lung disease and pulmonary disorders [33]. However, little is known about injury and host protective mechanisms in A-T lung disease. Furthermore, dysphagia in patients with A-T is a well known clinical symptom [25]. Similar to lung disease, this problem appears to be progressive and the assumption that swallowing function worsens with age is consistent with the progression of neurodegeneration and the bulbar impairments associated with A-T [5]. The impact of cellular and clinical factors on A-T lung disease is unknown so far. In our study, we investigated a pathogen-independent damage in order to determine differences in host mucosal inflammatory and repair mechanisms in *Atm*^-/-^ mice in comparison to control mice. Interestingly, *Atm*^-/-^mice did not show any clinical symptoms except for smaller size at birth and a slower growth rate compared to the wild-type littermates [34]. However, when challenged with hydrochloric acid, causing a disruption in the integrity of the airway epithelial barrier, we showed that *Atm*^-/-^mice exhibit greater severity of clinical symptoms and mortality rates as well as airway recruitment of peripheral leukocytes, and mount an even stronger immune response characterized by inflammatory biomarkers compared with wild-type mice. This is in accordance with the finding that A-T patients do exhibit elevated serum IL-8 levels, reflecting a systemic inflammation contributing to disease phenotype [35]. Since weights of the Atm^-/-^mice were less in comparison to wild-type mice, initial application of 50 μl hydrochloric acid may have shown a high mortality rate in *Atm*^-/-^mice due to a higher volume per body weight. So we adjusted the application of hydrochloric acid volume to the body weight of the mice.

Epithelial damage, PMN recruitment and activation of host protective mechanisms are early events in acute mucosal inflammation and ALI/ARDS. In this study, we confirmed the pivotal role of ATM in decreasing the severity of HCl acid-initiated ALI. ATM deficiency favored disruption of epithelial barrier integrity after ALI, as indicated by histological findings, and BALF biomarkers, namely IL-6 and TNF-α. In addition, we found an elevation in lung resistance and reduction in tissue compliance before intratracheal administration of HCl that was not altered after ALI. This data is in accordance with pulmonary function testing (PFT) in A-T patients 12–20 years of age, showing a mixture pattern of obstructive and restrictive lung disease [21,24,36]. The current study explored for the first time the inflammatory response of airway mucosa after injury by HCl. In accordance with human studies showing early structural changes, particulary bronchiectasis and consolidation [37], our *Atm*^-/-^mice showed decreased compliance and increased obstruction before musosal injury occurred. This is highly suggestive for the role of ATM for epithelial cell integrity homeostasis.

In other murine models, e.g. an experimental model of colitis, or acute inflammation, ATM deficiency also increases production of pro-inflammatory mediators and regulates leukocyte trafficking to inflammatory sites [38]. Here, ATM deficiency may have contributed to an increased inflammatory response confirmed by levels of several pro-inflammatory mediators in the injured lung.

On the one hand, neutrophilic invasion into the airways is critical to clear pathogens from the site of infection and suppression of the inflammatory response may increase the risk of infection-related adverse events, on the other hand, neutrophilic activation can cause bystander tissue damage that contributes to the pathogenesis of ALI/ARDS [39]. To this end, inhibition of PMN function in animal studies attenuates lung injury induced by models of gastric acid aspiration [40,41]. In this context our data suggest that ATM is a potent regulator of mucosal repair and can promote an array of protective responses for lung catabasis after mucosal injury.

Together, the differences of mucosal immune response and mucosal repair mechanism in *Atm*^-/-^ mice point to the clinical impact of repetitive mucosal injury by non-pathogen associated mucosal damage in order to explain decline in lung function in our A-T patients. Furthermore, ATM may be a critical immunoregulatory factor dampening the deleterious effects of acute HCl-induced inflammation, being mandatory for systemic genomic stability and homeostasis of the lung epithelial barrier.

## Conclusions

Even though the results of our study are more descriptive, this is the first study showing aggravated non-pathogen damage response after airway mucosal injury in an in vivo AT- mouse model.

*Atm*^-/-^mice are more sensitive to HCl-induced acute inflammation than control mice, especially during remission (24 hours) and up to one week after lung injury, showing lack of repair of incurred damage. ATM therefore can be inferred to play a critical role in immunoregulation after airway mucosal damage. Further investigations about the role of ATM in achieving homeostasis after airway mucosal injury may help to understand the pathogenesis of A-T lung disease.

## Competing interests

The authors declare that they have no competing interests.

## Authors’ contributions

OE and RS designed the protocol. OE, SK, SV, CH, RPD and JP performed the experiments. CD and SW made the histological studies. All authors analyzed and discussed the data. OE and SZ wrote the manuscript. All authors read and approved the final manuscript.

## Pre-publication history

The pre-publication history for this paper can be accessed here:

http://www.biomedcentral.com/1471-2466/14/93/prepub
